# Carbonation and Chloride Ions’ Penetration of Alkali-Activated Materials: A Review

**DOI:** 10.3390/molecules25215074

**Published:** 2020-11-01

**Authors:** Xuanhan Zhang, Kaidi Long, Wei Liu, Lixiao Li, Wu-Jian Long

**Affiliations:** Guangdong Provincial Key Laboratory of Durability for Marine Civil Engineering, Shenzhen Durability Center for Civil and Transportation Engineering, College of Civil and Transportation Engineering, Shenzhen University, Shenzhen 518060, China; zhangxuanhan2019@email.szu.edu.cn (X.Z.); longkaidi2019@email.szu.edu.cn (K.L.); liuwei@szu.edu.cn (W.L.); lilixiao@szu.edu.cn (L.L.)

**Keywords:** alkali-activated materials, durability, carbonation, chloride ions attack, enhancing method

## Abstract

Alkali-activated materials (AAMs) are widely recognized as potential alternatives to ordinary Portland cement (OPC) due to their lower carbon footprint. However, like OPC, AAMs can also generate some durable problems when exposed to aggressive environments and the mechanisms and possible improvements are still not fully clear in existing investigations. Furthermore, the corrosion mechanisms of AAMs are different from OPC due to the discrepant reaction products and pore structures. Thus, this study’s aim is to review the chemical reaction mechanisms, factors, and mitigation methods when AAMs are attacked by carbonation and chloride ions, along with a summative discussion regarding instructive insights to durable problems of AAMs.

## 1. Introduction

With increasing construction demand due to the rapid development of economies, the production of ordinary Portland cement (OPC) consumes large amounts of resources accompanied by a great deal of CO_2_ emission. Accordingly, the pursuit of building materials with a low carbon footprint is intriguing worldwide [1,2]. In particular, among the promising alternative binders, alkali-activated materials (AAMs) which exhibit superior durability and environmental performance over OPC received most of the attention in the past two decades and have been studied extensively by much research [2,3,4,5,6].

**Figure 1:**
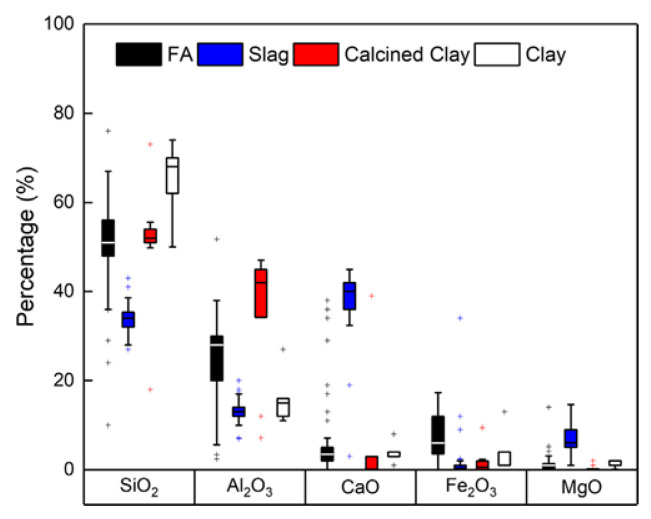
Average chemical composition of all aluminosilicate alkali-activated material (AAM) precursors reviewed in the literatures [12].

**Figure 2:**
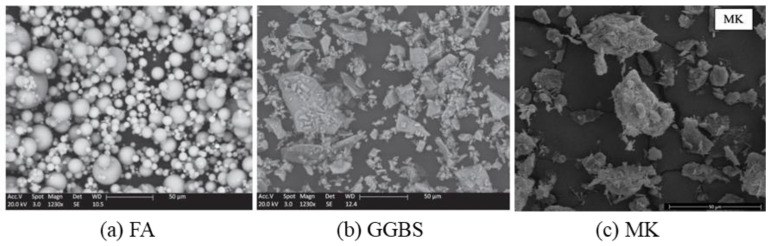
Scanning electron microscope (SEM) images for (**a**) fly ash (FA), (**b**) granulated blast furnace slag (GGBS) [16], and (**c**) metakaolin (MK) [17].

Generally, AAMs are produced through the polymerization reaction between solid aluminosilicate sources (precursors) and alkaline activator solution. Granulated blast furnace slag (GGBS) [7,8], fly ash (FA) [9,10], and metakaolin (MK) [11] are the major precursors utilized in studies about AAMs [7,12,13,14,15]. Figure 1 and Figure 2 are the chemical composition and scanning electron microscopy (SEM) images of these precursors, respectively [12,16,17].

The types of alkaline activators also play an important role in AAMs. Commonly used activators include potassium hydroxide (KOH), sodium hydroxide (NaOH), and waterglass (Na_2_SiO_3_) solutions. Besides, sodium carbonate (Na_2_CO_3_), sodium sulfate (Na_2_SO_4_), and calcium hydroxide (Ca(OH)_2_) have also been used as activators in several literatures [18,19]. The reaction mechanisms and products of AAMs are different from OPC systems. Calcium-silicate-hydrate (C–S–H) is the main hydration product in OPC systems, while the main reaction gels in AAMs are calcium-aluminosilicate-hydrates (C-A-S-H) and sodium-aluminosilicate-hydrate (N-A-S-H). Additionally, crystalline phases such as hydrotalcite and other crystalline phases also coexist in AAMs [20,21,22]. Furthermore, AAMs exhibit improved durability due to the discrepancy of the reaction phases compared to OPC. For example, the hydrotalcite-type phase in AAMs shows strong chloride immobilization capacity, which makes contribution to the higher resistance against chloride penetration of AAMs [23,24,25,26].

Although AAMs have been demonstrated to have superior durability properties, the underlying mechanisms and possible improvements are still not fully clear in existing investigations [1,2,3,4]. Among these unsolved issues, it is crucial to understand the long-term durability of AAMs against corrosion of steel reinforcement. Furthermore, it is well-accepted that the corrosion of steel reinforcement induced by chloride ions is one of the main causes accounting for the degradation of concrete structures. Furthermore, carbonation in the binding matrix is showed to decrease the pH in the pore solution. Moreover, the decreased pH value caused by carbonization is reported to combine with the increased concentration of chloride ions, which further contributes to the break of passive layers and induce the corrosion of steel reinforcements [27]. As a matter of fact, carbonation and chloride ions’ penetration of AAMs are the critical durability factors which contribute to the degradation of concrete structures [6]. Therefore, it is critical to understand the mechanisms and the factors affecting carbonation and chloride ions’ attack in AAMs.

Carbonation is one of the serious durability problems of concrete, which mainly accelerates the corrosion of steel reinforcement during the carbonation process due to the decrease in the pH of pore solution. Furthermore, some hydration products degraded as well. From the available research, AAM concrete exhibits lower resistance to carbonation than OPC concrete [28,29]. It is widely accepted that the main factors affecting carbonation of AAMs include the type of precursors, the activators’ composition, concentration, and type of alkali activators, silicate modulus, carbonation conditions, natural carbonation, and accelerated carbonation (the biggest difference of which is the difference in CO_2_ concentration). In addition, major influences and performances in cement as a result of carbonation are reflected in the following areas that include carbonation depth, mechanical strength, and pore structure, and the factors affecting carbonation in AAMs will be clearly introduced in Section 2.

In general, chloride ions from external environments penetrate into concrete by absorption [30] and diffusion [31], and the chloride ions cause corrosion of steel by a depassivation process [32]. Compared to OPC systems, the factors affecting chloride penetration in AAMs system are quite different, including the precursors system and its physical characteristics (crystallinity and fineness) and chemical properties (calcium content, aluminum content, and magnesium content), activators’ composition, silica modulus, curing conditions (temperature, humidity, and curing time), and water-to-binder ratio (w/b). Moreover, exploratory studies have demonstrated that pore structure and porosity could be influenced by these factors, which is believed to decrease chloride penetration [33,34,35,36]. Moreover, it is crucial to recognize that the chloride-binding capacity in AAMs is reportedly higher than in OPC structures owing to the additional gelation of binders, including C-A-S-H, N-A-S-H, and the hydrotalcite-type phase [37,38,39,40,41,42,43,44,45]. More C-A-S-H gel is reported to reduce the porosity and chloride penetration, and additional gelation of N-A-S-H gels also has positive effects on the chloride penetration resistance in AAMs due to the reduction in pore volume. Furthermore, the hydrotalcite-type phase has been demonstrated to mainly make a contribution to the higher chloride-binding capacity in AAM structures [25,26]. The factors affecting the penetration of chloride ions in AAMs will be clearly discussed in Section 3.

In this paper, the durability performances of AAMs, primarily including carbonation and chloride-resistance capacity, are reviewed and clearly discussed, and the objective of this work is to review the factors affecting the carbonation and chloride transport of AAM structures. Furthermore, the reinforcing methods for AAMs against carbonation and chloride penetration will be introduced in Section 4.

## 2. Common Perspectives

It is well accepted that a lot of literature has been made on understanding AAM systems. Moreover, carbonation and chloride ions’ attack are the main causes accounting for the degradation of concrete structures, mainly due to the corrosion of steel reinforcement. Therefore, this paper reviews and further discusses the factors affecting carbonation and chloride attack in AAM systems. Section 3 and Section 4 review the factors affecting carbonation and chloride ions’ attack in AAM systems, respectively. Furthermore, the enhancing methods for AAMs against carbonation and chloride ions’ attack are clearly proposed in Section 5.

The factors and reinforcing methods for AAMs against carbonation and chloride ions’ attack should mainly consider properties of precursors, activators’ composition, and silica modulus. The increased fineness and reduced crystallinity degree of precursors are illustrated to accelerate dissolution of the precursors and promote the activating process. Additionally, the chemical properties of precursors will affect the formation of binder gels and additional crystalline phases, which influences the carbonation and chloride resistance. Higher alkali content and increase in silica modulus tend to improve chloride- and carbonation-resistance in AAMs owing to the reduction in porosity and the reduced pore sizes. However, beyond a certain amount, it will decrease.

Additionally, CO_2_ concentration used in carbonation testing has a huge impact on the results and requires special attention, and currently, the more stable test condition is less than 1% CO_2_ concentration. Moreover, curing conditions and water-to-binder ratio also should be considered in reinforcing the chloride resistance of AAMs. The curing conditions can also influence the chloride resistance of AAMs systems. For example, the resistance to chloride ions can be improved by an extended curing time and increasing relative humidity. A higher curing temperature leads to decreased porosity and chloride penetration due to the improved hydration process and increased formation of new compounds of hydration. However, continued elevated temperature curing could show a strength loss in the long-term strength development. The following sections will clearly discuss the factors affecting carbonation and chloride ions’ attack of AAMs.

## 3. Carbonation

### 3.1. Carbonation Mechanism in AAMs

**Figure 3:**
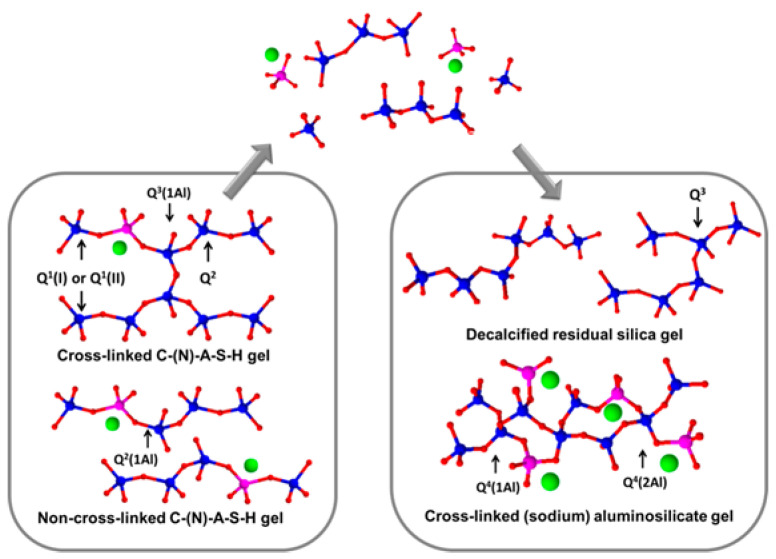
Structure change of slag-based AAMs during carbonation; blue balls: Si; pink balls: Al; red balls: O; green balls: Na [47].

The carbonation of concrete is a serious durability issue which refers to a neutralization reaction. During the carbonation process in which CO_2_ in air dissolves in pore solution and further produces [CO_3_]^2−^ ions, these ions react with the hydration products of AAMs, and the structure change of AAMs during carbonation is as shown in Figure 3. Consequently, the carbonation reaction can decrease high alkalinity in the pore solution and damage the passivation film of the steel bar in concrete and accelerate the corrosion of steel bars [46].

**Figure 4:**
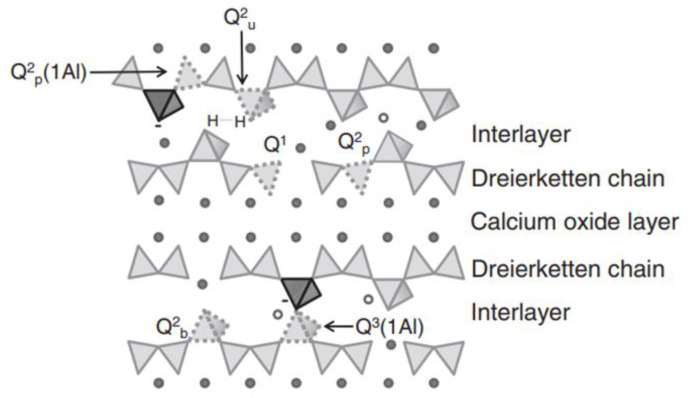
Schematic structure of calcium-aluminosilicate-hydrates (C-A-S-H). Interlayer includes calcium ion, water, alkali, or negative charge; dreierketten chain includes SiO^4–^ and AlO^4-^; Q^n^_(mAl)_ indicates the number of adjacent Si and Al; b and p indicate the bridging position and pairing position, respectively [53,54].

In general, AAMs are less resistant to carbonation than OPC [48,49,50,51,52] because of the presence of calcium hydroxide (CH) as the buffer material reacting with carbonate ions to consume some of the introduced carbon dioxide. However, CH is not present in AAMs, resulting in a faster pH drop in the cement pore solution and more rapid dissolution of C-(A)-S-H under the carbonation process, which, therefore, leads to more severe carbonation. In addition, the carbonation mechanism of AAMs is more complex than that of OPC. Considering the differences in the properties of raw materials, AAMs can be divided into high- and low-calcium systems, of which the carbonation mechanism is diverse. For the high-calcium systems, the major gel is C-(A)-S-H, which is similar to Figure 4.

**Figure 5:**
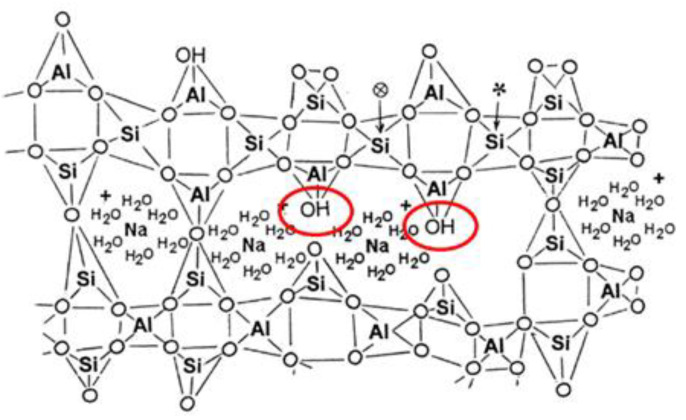
Schematic structure of sodium-aluminosilicate-hydrate (N-A-S-H) [48].

In this case, the ingress of CO_2_ can change the solid–liquid equivalent curve of Ca through the formation of CaCO_3_, promoting the decomposition of C-(A)-S-H gel. However, the major product in low-calcium systems is N-A-S-H, which is similar to Figure 5.

Its carbonation is mainly to reduce the high alkalinity and form sodium carbonate with a high concentration in the pore solution [55,56], but the structure of N-A-S-H gel does not change. In addition to alkali-activated precursors affecting the carbonation properties, different concentrations, types of alkali activators, and carbonation condition could also lead to different hydration products and carbonation properties [57].

### 3.2. Factors Affecting Carbonation in AAMs

#### 3.2.1. Type of Precursors

Numerous investigations have found that the precursor composition in AAMs [58,59,60,61,62,63,64] has an obvious influence on the resistance to carbonation. This is mainly attributed to differences in the content of calcium, silicon, magnesium, and aluminum in the precursors and, thus, variations in the hydration products.

Bernal et al. [65] found that the addition of metakaolin can increase the setting time of AAM paste and reduced susceptibility to carbonation. In another study, after accelerated carbonation for 540 h at the same humidity, an increased content of metakaolin in AAM concrete led to a reduction in carbonation depth [66], which was attributed to the fact that the addition of metakaolin increased the amount of MgO in precursors. Further, Bai et al. [67] added active MgO to the alkali slag system and concluded that the addition of active MgO facilitated the form of hydrotalcite-like compounds and slowed the degradation of C-(A)-S-H gel, thereby improving the carbonation resistance of AAMs. In addition, slag-based AAMs with higher magnesium content had increased resistance to accelerated carbonation [68] because the increasing magnesium content promoted the formation of a stable Mg-stabilized amorphous calcium carbonate (ACC) phase and reduced the degradation of C-(N)-A-S-H gels [69].

However, the addition of fly ash (FA) can lead to more severe carbonation of slag-based AAMs. Nedeljković et al. [70] probed the influence of raw material type on carbonation rate using different ratios of FA and GGBS mixes. Results show that the replacement of FA higher than 50 wt.% had a greater carbonation rate than that less than 50 wt.% due to the fact that FA had lower activity and required more alkalis to stimulate its activity. Consequently, more OH- ions were consumed in the hydration process, resulting in lower pH values of the pore solution and thus decreased buffering capacity during carbonation. Park et al. [71] also concluded that the carbonation area measured by phenolphthalein became larger as the content of FA increased in FA/BFS mortar.

In addition, the carbonation of AAMs with the mix of natural pozzolan and GGBS as raw materials was studied. Salazar et al. [72] claimed that the pH value of alkali-activated concrete containing natural pozzolans and GGBS could stabilize to about 10.5 under accelerated carbonation and it was higher than the pH (pH > 9) at which the rebar passivation film is destroyed [48]. Considering the fact that phosphorous slag is also a potentially alternative material, You et al. [73] studied the carbonation resistance of AAMs using phosphorous slag and FA as raw materials, and demonstrated that the carbonation resistance was similar to OPC.

Differences in precursors not only have a significant effect on the depth of carbonation but also on the change in strength after carbonation. Bernal et al. [74] claimed that the compressive strength of AAMs produced with GGBS and metakaolin decreases significantly after carbonation and the compressive strength is linearly related to the carbonation depth. Similarly, under an accelerated carbonation environment, a decrease in the pH of AAS, decalcification of C-(A)-S-H, and increase in porosity could be observed. Consequently, the decrease in compressive strength of the sample after carbonation was attributed to a larger diffusion coefficient and the structure became less compact due to carbonation [75]. There are two explanations for the decrease in compressive strength of AAMs after carbonation. One is that the decrease in compressive strength is due to the small amount of calcium carbonate, which cannot compensate for the decrease in strength caused by the degradation of C-S-H [76]; the other explanation is that although the low Ca/Si of C-S-H is more durable, it breaks down faster and the cohesion decreases once it starts to degrade [77].

However, a subset of researchers have come up with the different results. Nedeljkovic et al. [78] observed that there was not a constant trend in the compressive strength of AAMs after carbonation and there was both uptrend and downtrend in the compressive strength during carbonation. Salazar et al. [72] used a mixture of natural pozzolan (70%) and granulated blast furnace slag (30%) as raw material; the change trend in the compressive strength of the prepared samples was found to fluctuate under 48 weeks of carbonation, and all compressive strength increased compared to the initial values, with the most rapid increase in compressive strength during the first 4 weeks.

In conclusion, low-calcium AAMs (such as FA and MK) have a deeper carbonation depth than high-calcium AAMs (such as slag-based AAMs), and metakaolin-based AAM concretes are less resistant to carbonation than FA-based AAM concretes [79]. The different strength developments of carbonated AAMs may be attributed to the type of precursors.

#### 3.2.2. Activators Composition

It is known that AAMs are manufactured by aluminosilicates and alkaline activator [2,3,5,80]. As one of the important components in the concrete structure, reinforcing steel in concrete generates a passivation film in an alkaline environment and its stability in AAM cements is most dependent on the concentration of alkali activators [81,82]. In particular, the types of alkali activators have important implications for reaction kinetics, the properties of the binders, and phase evolution [83,84,85]. Therefore, the effect of the concentration and type of alkali activators on the carbonation resistance of AAS is discussed.

With increasing concentrations of alkali activators, the carbonation depth would be reduced in mortars of AAS, regardless of whether the raw material is a single slag or a mixture of slag and metakaolin [65,86]. Shi et al. [57] compared different alkali dosages (6% and 8%) of granulated blast furnace slag with a constant silicate modulus, and the results showed that the carbonation depth and the porosity of AAM mortars decreased after carbonation as the alkali dosages increase, meaning that an increase in the alkali dosages improved the carbonation resistance of AAMs. Song et al. [87] compared the carbonation depths of slag-based AAMs with different alkali content and OPC under different carbonation times, and the results showed that the carbonation depth of GGBS with alkali content of 6 wt.% was much greater than that of OPC, while the carbonation depth of AAMs with an alkali content of 14 wt.% was similar to that of OPC under a long time. Even the carbonation depth of AAMs was less than that of OPC after 7 days of carbonation, which meant that increasing the concentration of alkali activators could increase the carbonation resistance of AAMs to the same level as that of OPC. This phenomenon can be attributed to the fact that the hydrated extent of AAMs becomes higher as the concentration of the alkali initiator increases, leading to a denser structure [57,87].

However, it is not constant that the higher the alkali concentration in all AAM systems, the lower the degree of carbonation. Conversely, Navarro et al. [88] used water glass as activator, and the increased concentration of alkali activator led to an increase in the carbonation depth of SiMn slag-based AAMs. When using coal-based synthetic natural gas slag as a raw material and NaOH as an alkali activator, it was concluded that excessive alkali concentrations caused significant carbonation [89].

In addition to the influence of concentration, the type of alkali activator is not ignored. Bai et al. [67] incorporated MgO into slag-based AAMs and found that the carbonation depth was lower when NaOH was used than Na_2_SiO_3_ but the strength of the carbonated cements increased slightly when Na_2_SiO_3_ was used. Ye et al. [90] took five different types of alkali activator (NaOH, Na_2_SO_4_, Na_2_CO_3_, KOH, and K_2_CO_3_) and found that compared with NaOH and Na_2_CO_3_, the carbonation performance of the slag-based AAMs with Na_2_SO_4_ was relatively poorer. Furthermore, it was also found that independent of the curing temperature, slag-based AAMs with sodium-containing activators have superior resistance to carbonation than those with potassium-containing activators.

The impact of activator composition on the strength change trend after carbonation is listed in Table 1.

Song et al. [87] claimed that a higher concentration of alkali activator would contribute to a higher compressive strength of slag-based AAMs, but unfortunately, when the concentration of alkali activator is high, the compressive strength of slag-based AAMs would be lost more during the carbonation process. Navarro et al. [88] produced AAMs using different concentrations of NaOH and Na_2_SiO_3_ as activators, respectively, and found that the compressive and flexural strengths of all samples were increased after carbonation.

In general, the increase in alkali activator concentration is a beneficial trend for improving carbonation resistance in AAMs but not absolutely. When the alkali concentration exceeds a certain level, it does not increase the carbonation resistance effectively and may even bring side effects [93]. Furthermore, carbonation resistance of AAMs is better with a stronger alkali or alkaline salt as alkali activators.

#### 3.2.3. Silicate Modulus

When the alkali content and type of alkali activator are kept constant, the silicon modulus has a crucial influence on the composition of the final gel [94]. It has been found that the rate of hydration is faster when using NaOH as an alkali activator than when using a Na_2_SiO_3_ activator [76,95,96]. In addition, there is much research that used NaOH and Na_2_SiO_3_ as mixed activators. When the concentration and type of activator were the same, the change trend after carbonation was also very different with the difference of silicon modulus [57,97]. Therefore, the influence of the silicon modulus in the carbonation of slag-based AAMs is unquestionable.

Several studies have demonstrated that the carbonation resistance of AAMs increases as the modulus of silicon increases. Law et al. [98] found that the carbonation depths of samples are similar at silicate moduli 1.0 and 1.25 and are less at modulus 0.75, which is attributed to the lower porosity and denser structure of the sample at a higher silicon modulus. Shi et al. [57] conducted carbonation experiments using AAMs with silicon moduli of 0.5, 1.0, 1.5, and 2.0 and the results showed improved carbonation resistance of samples with increasing silicon modulus. Bernal et al. [92] compared AAMs with three different silicate moduli of 1.6, 2.0, and 2.4. The carbonation depth of the samples was found to be lower with the higher silicon moduli when the slag was used, indicating that an increase in the modulus of silicon is beneficial to reduce the degree of carbonation. Zhang et al. [97] also found that the carbonation rate was most rapid when the silicon modulus was 0 and the amount of CaCO_3_ produced from carbonation was greatest. When the silicon modulus is increased, the carbonation rate decreases significantly and the amount of calcium carbonate decreases, which was attributed to the change in porosity and pore structure. In particular, Zhang et al. [97] considered the comparison of different contents of slag/fly ash and found that although the carbonation mechanism of AAMs containing 20% slag was different from that containing 60% slag, there was the same trend where carbonation rates decreased with increasing silicon modulus.

However, it has been shown that NaOH-activated slag-based AAMs have better resistance to carbonation than Na_2_SiO_3_-activated slag-based AAMs [76]. In a document mentioned in this article [92], Bernal et al. also found that when the raw material for AAMs changed from slag to a mixture of slag and metakaolin, the trend of carbonation depth became contrary with the increased silicon modulus, which suggested that the trend in different precursors was not the same.

The contradiction between the two different trends above indicates that the trend of carbonation rate due to changes in silicon modulus is not clear and more research is needed to find out the reason in the future.

#### 3.2.4. CO_2_ Concentration

**Figure 6:**
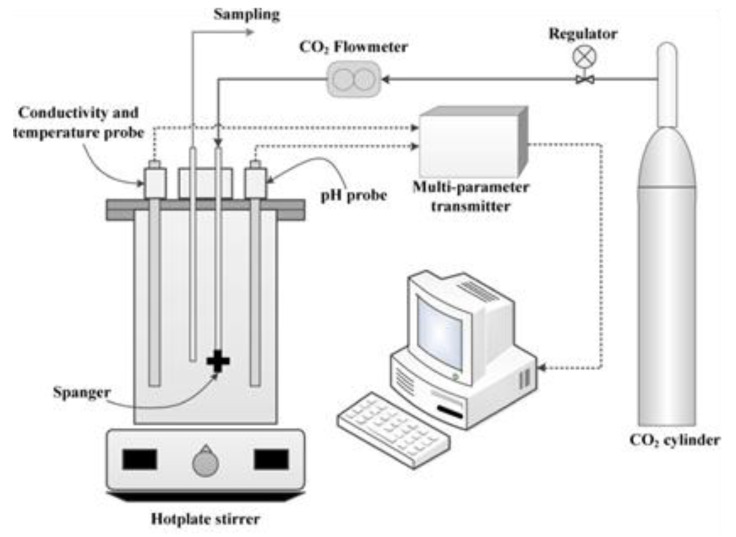
Schematic diagram of accelerated carbonation device [99].

The carbonation of AAMs has been generally studied by two types of test means—natural carbonation and accelerated carbonation. Accelerated carbonation, for which the apparatus is shown in Figure 6, refers to the increment in CO_2_ concentration and the curing temperature for shortening the research time. Here, the different conclusions from natural and accelerated carbonation are listed in Table 2 and the effect of CO_2_ concentration on carbonation of AAM cement is described below.

Several studies have reported that AAMs were less susceptible to carbonation than OPC under natural carbonation conditions [75,91,98,100,102], and this is not consistent with the results of many studies with accelerated carbonation tests [48]. AAMs made of GGBS and OPC were investigated under natural and accelerated carbonation conditions, respectively [100]. The results showed that the carbonate/bicarbonate equilibria was altered and more bicarbonate was produced under accelerated carbonation conditions with high concentrations of CO_2_, leading to the pH in the pore solution dropping below 10. The studies [48,93,100] came to the same conclusion. Similarly, Bernal et al. [74] claimed that the experimental results of AAMs under accelerated carbonation are not quite the same as those under natural carbonation. The result showed that the carbonation depth of slag-based AAMs under natural carbonation was lower than that under accelerated carbonation, indicating that the accelerated carbonation test method would underestimate the carbonation resistance of slag-based AAMs.

**Figure 7:**
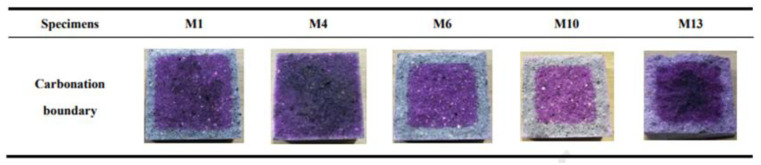
Schematic diagram of accelerated carbonation device [103].

There are a number of explanations for different results when the CO_2_ concentration varies. Bernal et al. [66] found that the CO_2_ concentration in the carbonation experiment had a great influence on the changes in the porosity of the sample. When the CO_2_ concentration was 1%, the pore size in the concrete increased linearly with carbonation time; however, when the CO_2_ concentration was 3%, the trend in porosity was not clear. This might indicate that different CO_2_ concentrations cause different changes in pores after carbonation, resulting in different trends. It can be also analyzed by means of carbonation test (such as carbonation depth test). The carbonation process of AAMs was composed of two steps, including the reactions in the alkali-rich pores and even the decomposition of the gel, while the reaction rate of these steps is greatly changed in the presence of high concentrations of CO_2_. Therefore, accelerated carbonation results from the general carbonation testing method (phenolphthalein test) are very different from the natural carbonation results because it can only examine the first step of the reaction [66]. Phenolphthalein test pictures are shown in Figure 7 [103].

Only the completely white area could be intuitively judged as a carbonation area, while the incompletely carbonation area is difficult to intuitively judge because the color changes little.

For AAMs which are more sensitive to CO_2_ concentration from [101], the influencing factors are more complicated, including differences in alkali content and raw materials. Therefore, in order to accurately estimate the impact of carbonation on the AAMs, the current recommended concentration of CO_2_ is 1% due to higher concentrations leading to obvious changes in the dissolved carbonate/bicarbonate equilibrium [103]; more details need to be further considered and more microscopic tests should be used to support the impact of carbonation.

## 4. Chloride Ions Attack

### 4.1. Chloride Penetration in AAMs

Generally, chloride ions coming from cement ingredients (e.g., sea sand) and marine environments can penetrate inside the porous system of structures through absorption, wicking, and diffusion effects [30,104]. In particular, the absorption effect refers to the capillary absorption driven by intermolecular forces, while the wicking effect is governed by the hydrostatic pressure. It is widely accepted that hydrostatic pressure can squeeze chloride ions into the cement matrix by pressure gradients applied to the cement surface [104]. Moreover, the diffusion effect derives from the concentration gradients between the external and internal environments. Exploratory studies demonstrated that the penetration depth and the rate of chloride ions in OPC and AAM concrete mainly depend on chloride diffusion coefficient (CDC) of the concrete mixtures [105]. Therefore, measurement of the CDC has become a crucial role during the process of assessment of reinforced concrete structures. Apart from the measurement of CDC, in general, other measurements of cement mixtures (e.g., rapid chloride migration (RCM) and rapid chloride permeability test (RCPT)) have also been used to evaluate the chloride resistance capacity in OPC and AAM systems.

The properties of hardened OPC and AAMs, such as pore solution chemistry, pore size distribution, porosity, tortuosity, and chloride-binding capacity, are suggested to affect the chloride transport in OPC and AAMs. Numerous studies [106,107,108,109] demonstrated that the factors, including physical and chemical properties of precursors, activators’ composition, and curing conditions, have impacts on porosity and pore size distribution, leading to the decrease in chloride penetration. For instance, the reaction products will be influenced by the physical and chemical properties of the precursors, affecting the porosity of the cement structures. Additionally, most research substantiated that higher alkali content and higher silica concentration can reduce chloride penetration in AAM binders, which is associated with reduced porosity [11,34,106,107]. Furthermore, the chloride-binding capacity of AAMs plays an important role in enhancing resistance to chloride penetration, and the chloride-binding capacity will be also affected by the composition of the AAM systems.

### 4.2. Factors Affecting Chloride Penetration in AAMs

#### 4.2.1. Type of Precursors

Generally, numerous studies demonstrated that the physical properties and chemical properties of precursors affect chloride penetration in AAM systems [110]. Table 3 lists the properties of precursors affecting the chloride resistance of AAMs.

It is well-accepted that the increasing fineness and reducing crystallinity degree of FA can lead to higher chloride-resistance capacity of AAMs. For example, Komljenovic et al. [113] substantiated that increasing the particle fineness of FA can accelerate its dissolution and activating process in AAMs, which further increased compressive strength and reduced the porosity; consequently, the chloride penetration of FA-based AAMs could be decreased. However, different to FA-based AAMs, it was demonstrated that the higher porosity and lower strength of slag-based AAMs are correlated with the increasing fineness of slag, attributed to the higher water demand and higher reactivity during its activating process [98,119]. Apart from slag and FA, calcined clays can be also utilized as the precursor in AAMs binders. More specifically, as one of the most used calcined clays in AAMs binders, MK has been reported to lower chloride penetration of AAMs, owing to the improved pore structure and reduced porosity [11,14,114,120]. Additionally, Weng et al. [111] illustrated an increase in mechanical strength from 55 to 74 MPa with increasing MK fineness from 15,670 to 25,550 m^2^/kg. Moreover, it is reported that particle fineness of the blended precursors also significantly affects the reactivity of the system [118,121].

However, the physical and chemical properties of precursors affect the chloride penetration in different mechanisms. It is well-accepted that the chemical properties of precursors have influences on the gelation of discrepant products in AAMs system, leading to improvement of micro-pore structures and the enhancement of chloride-resistance capacity. Generally, more C-A-S-H gel can reduce the porosity and chloride penetration in AAMs. Bernal et al. [114] and Ismail et al. [116] have demonstrated that the calcium content of precursors is a crucial factor which affects the mechanical performance and chloride-resistance capacity of AAMs since the ratio of Ca to (Al + Si) of C-A-S-H gel can lead to improvement on the degree of crystallization. In addition to the gelation of C-A-S-H, more N-A-S-H gels also have influences on the resistance to chloride penetration in AAMs due to the reduction in pore volume. Furthermore, it is reported that the content of calcium in precursors has effects on the production of N-A-S-H gels [112,116]. Winnefeld et al. [112] demonstrated that lower porosity is exhibited in low calcium FA-based AAMs than high calcium FA-based AAMs, owing to the high production of N-A-S-H gel. Moreover, the aluminum content of precursors affects the formation of N-A-S-H gel. Duan et al. [117] reported that increasing percentage of MK in the FA/MK-based AAMs is illustrated to decrease average pore size, which can be attributed to the gelation of more N-A-S-H gels deriving from the high aluminum content in MK. However, Ismail et al. [115] found that when increasing FA content, the volume of permeable voids is increased as a result of the excessive gelation of N-A-S-H gel, which reduces the resistance to chloride penetration and could induce corrosion of the rebar.

Furthermore, secondary reaction products, such as hydrotalcite-type phase and zeolite-type phase, are affected by the content of magnesium and aluminum of precursors. It is well-accepted that the formation of the secondary reaction products is shown to influence the chloride resistance capacity [26,27,122,123]. More specifically, numerous studies have demonstrated that the formation of hydrotalcite significantly enhances chloride-binding capacity, further strengthening the resistance to chloride attack [23,24,25,26]. For instance, Machner et al. [123] have demonstrated that the compound of dolomite fines consisting of magnesium and MK enhanced chloride-binding capacity and decreased chloride penetration due to the production of hydrotalcite, which was attributed to the large amount of magnesium provided by dolomite. However, Ye et al. [23] claimed that the potential of dolomite in enhancing the formation of hydrotalcite-type phase is highly limited in AAM binders owing to the restriction of the dissolution and reaction of dolomite with the absence of CH. According to the discussion above, it is recognized that the physical and chemical properties of precursors could affect the resistance to chloride attack of AAMs; more importantly, it is crucial to explore factors affecting the production of hydrotalcite in AAMs.

#### 4.2.2. Activators Composition

Alkali composition in activating solutions has intuitive influences on the gelation and formation of different aluminosilicate binder gels, which could affect the resistance capacity to chloride attack in AAMs [13,14,33,107,108,124]. Table 4 lists the compositions of activators affecting the chloride resistance of AAMs.

In general, the alkali content is stated as the molarity of metal hydroxide of activating solutions (e.g., 4M NaOH and 4M KOH) which can provide a lot of metal cations (e.g., Na^+^, Ca^2+^, and K^+^) and hydroxide anions during the process of activation. The alkali content also refers to the mass ratio of metal oxide to precursor (e.g., mass ratio of Na_2_O to slag) [12]. A higher alkali content makes contributions to facilitate the dissolution of precursors and accelerate the hydration of precursors, which further decreases chloride penetration in AAMs, owing to the reduction in porosity [13,14,34,124,129,130]. Hu et al. [106] reported that increasing the alkali content from 2% to 8% in slag/fly ash-based AAM mortars can reduce the porosity from around 16% to 10%, as well as decrease the chloride migration coefficient (10^−12^ m^2^/s) from 3 to 1.5, due to the accelerated dissolution of the precursors. Be et al. [114] also reported that decreased water sorptivity and chloride permeability are displayed with higher alkali content in slag/MK-based AAMs. Additionally, Za et al. [124] indicated that the density of fresh AAMs increased with higher NaOH molarity as a result of the faster dissolution of precursors and lower porosity. Moreover, Ma et al. [108] reported that the chloride penetration of AAMs could decrease with the increasing alkali content owing to the reduced porosity and pore sizes. Furthermore, the reduction in the chloride permeability and migration coefficients of slag-based AAMs results from the higher Na_2_O-to-slag ratio [36]. In addition, the improvement in microstructure development is elucidated with the higher alkali content, which could reduce chloride penetration in AAMs [11,33,34,35,36,125]. For example, Chindaprasirt et al. [125] suggested that higher NaOH concentration resulted in the enhancement of chloride resistance capacity and reduction in the CDC of AAMs due to the improvement of the pore structure. However, the higher alkali content may cause a reduction in resistance to chloride attack. Fang et al. [35] demonstrated that the large drying shrinkage and the deterioration in the resistance to chloride penetration of AAMs can be caused by the high alkali contents, which may associate with the micro-cracks induced by the high alkali molarity.

Although alkali content could refer to the quantity of elemental alkali metals in activators, different kinds of alkali metal cations (e.g., Na^+^, Ca^2+^, K^+^) in activating solutions have been proved to affect the reaction process, porosity, and chloride penetration in AAMs [18,33,131,132]. Xu et al. [120,132] reported that the activator made by KOH shows better reaction products during the activating process than NaOH, so the former has a lower porosity than the latter.

**Figure 8:**
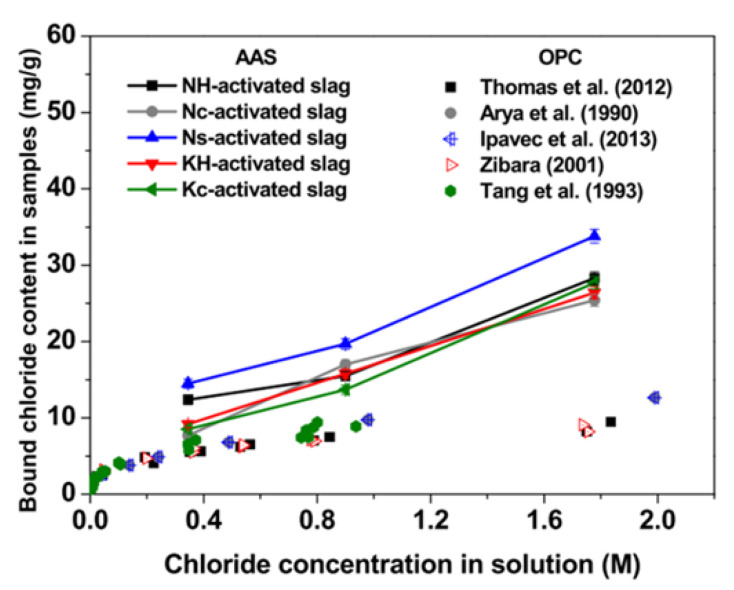
Chloride-binding isotherms of hardened slag-based AAMs pastes with various types of activator [18,134,135,136,137,138].

Moreover, it is attractive that the chloride-binding capacity of AAMs could be influenced by alkali content. Zhang et al. [7] found that reduced chloride-binding capacity of slag/fly ash-based AAMs is reported with increasing Na_2_O concentration, which is associated with a higher concentration of OH^-^ ions in the pore solution. Besides, it was also demonstrated that the composition of activators has influences on the chloride-binding capacity of AAMs [18,133]. As shown in Figure 8, Ye et al. [18] found that the highest chloride-binding capacity was demonstrated in sulfate-activated slag-based AAMs, which was related to the transformation of existing ettringite to Friedel’s salts.

#### 4.2.3. Silica Modulus

Apart from higher alkali content, increased chloride resistance has been also substantiated with the increment of silica content. Generally, silica modulus has been considered as the total silica amount in activating solutions; in addition, silica concentration has been defined as the Si/Al or SiO_2_/Al_2_O_3_ molar ratios in AAMs systems or the SiO_2_/Na_2_O ratio (denoted modulus, Ms) in the activating solutions [12,35,128]. Table 4 lists the silica modulus affecting the chloride resistance of AAMs.

In fact, exploratory literature demonstrated that reduction in chloride penetration is reported with higher available silica concentration in activating solutions. Moreover, the added silica from liquid Na_2_SiO_3_ solution shows better improvement to chloride resistance than powder sodium silicate [11,36]. It is suggested that added silica from liquid Na_2_SiO_3_ solution can decrease overall porosity and chloride resistance, attributed to the changes in the pore structure [36].

**Figure 9:**
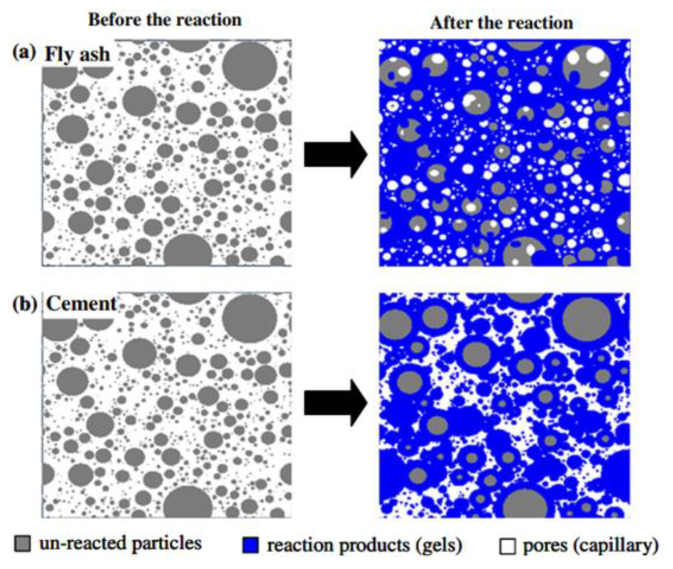
Formation of the microstructure in different systems caused by different silica modulus: (**a**) AAMs activated with a high silica modulus; (**b**) AAMs activated with a low silica modulus or cement paste materials no silica [108].

Moreover, experimental evidence has indicated that higher available silica in AAM systems, regardless of kind of precursor, not only led to a decrease in porosity and average pore sizes but also had a positive impact on chloride-binding capacity, which contributes to enhancing chloride resistance [11,35,106,107,108]. Hu et al. [106] substantiated that the compressive strength of AAM mortars can be increased with a higher silicate modulus due to the improvement of pore structure within the range of 10–104 nm, which enhances chloride resistance. In addition, Ye et al. [126] demonstrated that higher silica concentration in slag-based AAMs created reduced porosity and smaller pore sizes. Similar trends have also been certified by Ma et al. [108]. It has been substantiated that a significant reduction on water permeability is reported with higher silica content due to the finer pore system. As shown in Figure 9, the pores in Figure 9a are finer than those in Figure 9b, due to the additional formation of reaction products caused by a higher silica modulus [108].

Additionally, Hu et al. [106] demonstrated that increasing the silica modulus from 0 to 1.5 can reduced porosity by around 10%, due to the additional formation of reaction products caused by the higher silica modulus. More specifically, accurate experimental data illustrated that smaller pores can be distributed in a more homogenous pore system with Si/Al ratios greater than 1.65. Additionally, it is shown that a higher silica content in an AAMs system accelerates the hydration process. Me et al. [139] reported that the increase in silica content in AAMs causes a decrease in porosity due to the increasing hydration degree, therefore increasing the volume of C–S–H and enhancing the resistance to chloride penetration.

**Figure 10:**
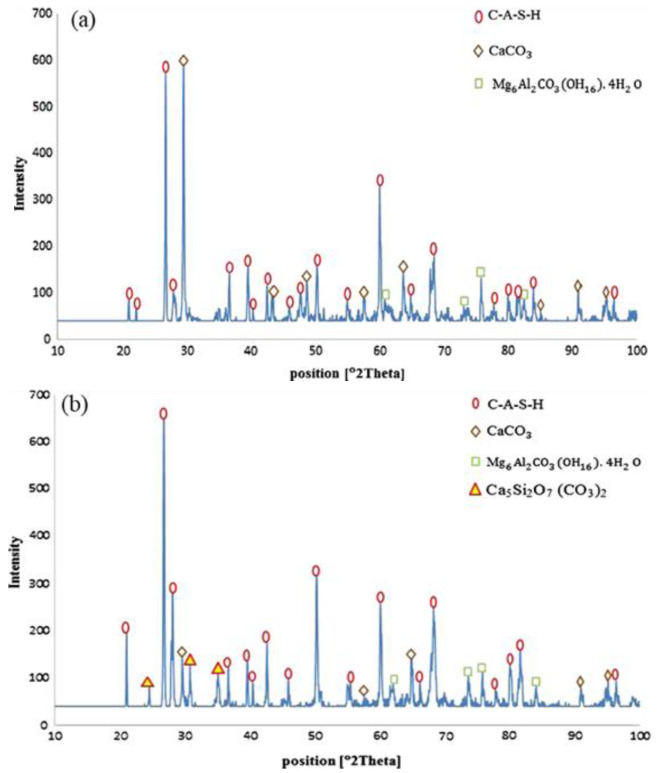
XRD pattern and phase analysis of (**a**) control sample and (**b**) nanosilica-containing slag-based AAM concrete [127].

In addition to the available silica content in an AAMs system itself, it is well-accepted that silica-rich substances (e.g., microsilica and nanosilica) show a significant improvement in chloride resistance as well. For example, Behfarnia et al. [127] observed that the addition of microsilica resulted in a better improvement in chloride-resistance capacity due to the increased production of C-A-S-H gels and the improved pore structures caused by the filling effect of micro particles in the matrix. Particularly, the addition of 3% nanosilica can enhance 28-day and 90-day compressive strengths by 12% and 11%, respectively. As shown in Figure 10, all peaks pertaining to C-A-S-H phase are on the rise with the presence of nanosilica [127].

Furthermore, Ra et al. [15] demonstrated that a significant improvement to mechanical properties and resistance to chloride penetration in AAM mortars can be found when nanosilica is added, which can be attributed to the increased formation of C-A-S-H gels and the improved microstructures.

#### 4.2.4. Curing Conditions

Besides the properties of precursors and the composition of activators, curing conditions (e.g., curing temperatures, curing time, and curing humidity) have been also demonstrated to affect chloride resistance in AAMs due to the changes in the kinetics of the reaction. Furthermore, experimental evidence has suggested that the changes in the kinetics of the reaction have impacts on improving the development of microstructures [23,129,140]. Table 5 lists the curing conditions affecting the chloride resistance of AAMs.

More specifically, the high-temperature curing in AAMs contributes to decreasing chloride penetration as a result of the reduced average pore sizes and reduced porosity [108,140,142,143]. Additionally, it is also reported that curing under a higher temperature can accelerate the dissolution of precursors and influence the overall generation of reaction products [14,90,144]. For instance, Teresita et al. [140] illustrated that porosity is decreased under higher temperature, which can be explained by the improved hydration process and increased production of new hydration substances. Moreover, the results are consistent with Ye et al. [90], who reported that the crystallinity of C-A-S-H increased with curing temperature; therefore, it has effects on decreasing porosity and further enhancing resistance to chloride penetration. Furthermore, an elevated curing temperature also yields increased electrical resistivity, compressive strength, and chloride resistance of FA-based AAM cement due to the lower volume of permeable voids [142]. However, there are some studies focusing on curing in AAMs at an ambient temperature, which is more practical on construction sites [23,130,140]. For example, Ye et al. [23] reported that a continuously elevated curing temperature can facilitate the transformation of strength-giving gel products into zeolite crystals in slag/MK-based AAMs systems, which can lead to a strength loss and a deterioration in the long-term strength development. In addition to high-temperature curing, it is well elucidated that a longer curing time has positive effects on the resistance to chloride ion attacks. Ma et al. [108] have reported that the water permeability of FA-based AAMs significantly decreased with a longer curing time, which is associated with the reduced total porosity and pore threshold diameter. Furthermore, experimental data demonstrated that the flexural strength, compressive strength, and chloride resistance of AAM mortar were enhanced with extended curing after 28 days, owing to reduced porosity and the increased gelation of binders [35].

Besides the curing temperature and curing time, the humidity in curing conditions has been illustrated to change the degree of reaction of the cement matrix, accordingly decreasing chloride penetration [145]. Serdar et al. [146] elucidated that improvements in strength and microstructure are demonstrated with a higher relative humidity of curing conditions in slag-based AAMs due to the higher hydration rate of slag grains. It is also reported that curing at a relative humidity of 80% and a temperature of 60 °C leads to improved durability of slag-based AAMs [141]. Differently from changing the curing humidity along the 28 curing days, coupling of wet and drying curing has been proved to affect the porosity of AAMs. More specifically, Mangat et al. [129] demonstrated that the wet/dry curing regime (3-day wet curing followed by dry curing at a temperature of 20 ± 2 °C) in AAMs results in the lowest pore volume and porosity compared with wet, dry, and wet/dry curing conditions; consequently, it leads to a significant enhancement in chloride resistance.

#### 4.2.5. Water-to-Binder Ratio

Apart from the factors mentioned above, the mechanical properties and workability of AAMs change with various water-to-binder ratios (w/b) due to the improved pore structures, which could further enhance resistance to chloride penetration [7,13,128,147,148].

In general, the w/b has been widely defined as the ratio of total liquid to total solids in the mixtures of AAMs. It is accepted that lowering the w/b can reduce chloride penetration owing to reduced overall porosity and decreased average pore sizes in AAMs [128]. Additionally, Be et al. [149] demonstrated that decreased water permeability and increased chloride resistance are shown with the low w/b, which is attributed to reduced porosity. This is similar to the research by Re et al. [150] where a low w/b in AAMs is reported to decrease water and chloride permeability due to decreased pore volume. Moreover, a related study has also proved that decreasing the w/b of slag-based AAM concrete is reported to reduce chloride penetration depth [13]. Furthermore, it has been substantiated that the w/b of FA-based AAM paste from 0.8 to 0.6 caused a decrease in porosity from 42.2% to 37.0% and in chloride penetration depth by 40–60% [9]. Although low w/b systems have exhibited excellent chloride resistance, the lowered water content may lead to air entrapment due to the increased viscosity of binders [148]. Meanwhile, it should be noted that the chloride-binding capacity of AAMs can increase with the w/b, which can be attributed to the OH- concentration in pore solutions [7]. Therefore, it is urgent to choose an appropriate w/b at first when designing a AAMs systems, which is tightly related with the resistance to chloride penetration.

## 5. Enhancement Methods for AAMs

### 5.1. Enhancement Methods for AASMs against Carbonation

Current research generally proves that the carbonation resistance of AAMs is worse than that of OPC, so it is essential to take corresponding measures to improve the carbonation resistance of AAMs. According to analysis of the carbonation mechanism of AAMs, there are generally two methods to improve the carbonation resistance of AAMs. One is to take certain measures to consume the carbonate ions which are produced from the dissolution of carbon dioxide in the pore solution. Another way is to add substances that can improve the alkali-activated concrete void structure and reduce the diffusion of carbon dioxide.

The main reason for the difference in carbonation between AAMs and OPC is that cement hydration produces Ca(OH)_2_, whereas the hydration of AAMs does not. Ca(OH)_2_ consumes carbonate ions from the pore solution, so the addition of a component that consumes carbonate ions effectively reduces the carbonation of alkali-activated cement. He et al. [151] introduced components that can react with carbonate ions into alkali-activated slag cement, including Ca(OH)_2_, Zn(OH)_2_, and ZnCl_2_, and determined the best ratio of the three components through experiments. Results showed that adding Ca(OH)_2_ was able to reduce the carbonation depth of slag-based AAMs more, by 33% in the 3-day carbonation test and by 45% after the 28-day carbonation test, and the other two components also reduced the carbonation depth to some extent.

Another way is to reduce the diffusion of carbon dioxide in alkali-activated cements. In particular, air-entraining agents are added to slag-based AAMs to introduce a large number of air bubbles smaller than 50 nm for reducing the later dry shrinkage of the cement and providing a better pore structure. Nedeljković et al. [152] argued that a better pore structure improved the carbonation resistance of slag-rich cements. The purpose is to react with the AFt or AFm produced by the hydration of alkali-activated slag cement to increase the crystallinity of the hydrated product [151]. Wang et al. [68] found that the pore size of high-Mg slag-based AAMs changed less during carbonation than low-Mg AAS and that the increase in Mg content led to a decrease in the loss of gel pores and an increase in the amount of large pores in AAMs. In addition, shrinkage cracking also affects the degree of carbonation of AAMs. Bilim et al. [153] investigated the effect of shrinkage-reducing (SHR) as well as superplasticizing and set-retarding admixtures (SSR) on the degree of carbonation of slag-based AAMs and showed that SHR can slightly reduce the carbonation depth of slag-based AAMs, while SSR has no effect on the carbonation of slag-based AAMs. Palacios et al. [77] also concluded that vinyl polymer and shrinkage-reducing admixtures did not significantly improve the carbonation resistance of alkali-activated slag cements. Behfarnia et al. [4] added silica fume to slag-based AAMs in order to improve carbonation resistance. As the content of silica fume increased, the carbonation depth of the sample gradually decreased. In particular, the carbonation depth of slag-based AAMs containing 15 wt.% silica fume reduced by 32%, which is similar to the result obtained by Duan et al. [154] by adding 5% of nano-TiO_2_ in fly ash-based AAMs. However, the former method was more economical.

At present, the carbonation resistance of AAMs is mainly improved by adding chemical admixtures and replacing some of the precursor materials, but most of them are only at the research stage. The next step should be to improve the possibility of its engineering application.

### 5.2. Enhancement Methods for AAMs against Chloride Penetration

As previously discussed, chloride-induced corrosion is another widely known cause accounting for the degradation of AAM concrete systems. Current literature has reported that resistance to chloride attack in AAMs is mainly affected by four factors, including the properties of precursors, composition of activators, curing conditions, and w/b. Moreover, these factors affect chloride transport through different mechanisms. Hence, reinforcing methods for AAMs against chloride attack should be considered via the four parts.

Numerous studies have demonstrated that enhancing chloride resistance is associated with the improved properties of the precursors. For example, when designing an AAM system, increased fineness and lowered crystallinity of precursors are chosen to reduce the chloride penetration owing to the accelerated activating process in AAMs [113,119,155].

Furthermore, it is demonstrated that the additional production of C-A-S-H gels can decrease porosity and water absorption, further reducing chloride penetration. More importantly, the additional formation of C-A-S-H and hydrotalcite-type phase exhibits excellent chloride-binding capacity, which can reduce free chloride ions in pore solutions [24,25,26,156,157]. Furthermore, modifications in the chemical properties of precursors (e.g., calcium content, aluminum content, and magnesium content) can affect the gelation of hydration products, such as C-A-S-H gels, N-A-S-H gels, and hydrotalcite-type phase [158]. For instance, adding Supplementary Cementitious Material (SCMs) or other substances which contain more aluminum content (e.g., MK) and magnesium content (e.g., dolomite) in AAM systems can contribute to the gelation of the hydrotalcite-type phase. Furthermore, the low calcium content of precursors (e.g., low-calcium FA) in AAMs can create more N-A-S-H and a lower porosity than a high calcium content in AAMs.

In addition, the changed composition of activators can also improve the resistance to chloride ion attacks of AAMs. It is well illustrated that a higher alkali content of activators by increasing the molarity of metal hydroxide (e.g., NaOH and KOH) of activating solutions can create denser microstructures owing to the rapid dissolution of precursors and the accelerated kinetics reaction process [36,108,124]. Moreover, it is elucidated that a higher silicate modulus (e.g., increasing the SiO_2_/Na_2_O ratio in activators or adding micro/nanosilica in systems) of AAMs can decrease the porosity caused by the higher C-A–S–H volume, which has a positive effect on enhancing the resistance to chloride attacks [126,127,139,158,159,160,161].

Moreover, the resistance to chloride ion attacks of AAMs can be also achieved by changing curing conditions. Numerous studies have substantiated that decreased chloride penetration is revealed with high-temperature curing on AAMs due to the accelerated dissolution of precursors, which contributes to reduced average pore sizes [108,140,142,143] and the overall creation of reaction products. In addition to high-temperature curing, it is well-demonstrated that a wet curing condition significantly decreases chloride penetration in AAMs, which is associated with the increased gelation of binders [129,141,147].

## 6. Prospects

At present, most of the research discussed the deterioration of AAMs under a single carbonation condition, but this was not consistent with the actual carbonation process. More studies are needed to further explore the impact of carbonation under a multi-coupling environment to better meet the actual conditions. Furthermore, previous investigations have demonstrated that the formation of hydrotalcite-type phase is reported to enhance the chloride resistance capacity of AAMs due to its strong chloride-binding capacity. However, the factors affecting the formation of hydrotalcite-type phase have not studied clearly. Moreover, AAM systems show superior chloride-resistance to OPC systems, but the mechanism of chloride binding in AAMs is not clear yet.

## Figures and Tables

**Table 1:** Activators composition and silica modulus affecting carbonation resistance of AAMs.

Year	Precursor(s)	Activators	Alkali Concentration (wt.% Na_2_O)	Silica Modulus	Compressive Strength	Ref.
2001	Slag	NaOH + Na_2_SiO_3_	2%	0.75	Decreased	[75]
2006	Slag	NaOH + Na_2_SiO_3_	1%	0	Increased	[91]
			1%	1.2	Decreased	
2010	Slag	NaOH + Na_2_SiO_3_	5%	1.6/2.0/2.4	Decreased	[92]
2014	Slag	Na_2_SiO_3_	3/5/7%	—	Decreased	[87]
2015	Slag + MK	NaOH + Na_2_SiO_3_	9.1–14.5%	—	Increased	[65]
2017	Slag	NaOH + Na_2_SiO_3_	—	0/0.5/1.0/1.5	Decreased	[76]
2018	Slag	NaOH + Na_2_SiO_3_	6/8%	0–2	Decreased	[57]
2020	Slag + NP	NaOH + Na_2_SiO_3_	—	1.1	Increased	[72]
2020	Slag + POFA + RHA	NaOH	4.5%	0	Decreased	[49]
	Slag + FA + RHA	NaOH	4.5%	0	Decreased	
2020	SiMn Slag	NaOH	3.0 /3.5 /4.0%	0	Increased	[88]
		Na_2_SiO_3_	4.0/4.55/5.0%	1.0	Increased	

NP = natural volcanic pozzolan; POFA = palm oil fuel ash; RHA = rice husk ash.

**Table 2:** Different conclusions from natural and accelerated carbonation of AAMs.

Year	Precursor(s)	Activators	CO_2_ Concentration	Carbonation Time	Conclusion	Ref.
2012	Slag	NaOH+ Na_2_SiO_3_	Natural/7%	—	A linear relationship between the natural carbonation depth and carbonation degree in 7% CO_2_	[100]
2014	Slag	NaOH+ Na_2_SiO_3_	Natural	7 years	Carbonation depth in natural carbonation was lower than predicted depth with accelerated carbonation	[74]
2015	Slag + MK	NaOH+ Na_2_SiO_3_	1%	1000 h	Pore size increased with carbonation time	[66]
			3%	1000 h	Pore size change trend was irregular	
2016	MK	Na_2_SiO_3_	Natural/50%	365 days	50% CO_2_ carbonation test results cannot represent natural carbonation results	[48]
2017	Slag + HPA+ FA	NaOH+ Na_2_SiO_3_	Natural/1%	500 days	The formation of bicarbonates is more than carbonates in 1% CO_2_	[93]
2020	Slag	NaOH	Natural	4 months	Type of CaCO_3_ produced by carbonation under natural carbonation was related to precursor.	[90]
2020	Slag	NaOH	3%	140 days	Total porosity and average pore size decreased	[101]
		Na_2_SiO_3_	0.03%/3%/20%	140 days	Carbonation depth with 3% CO_2_ was twice that of 0.03%; total porosity decreased	

HPA: high-performance ash.

**Table 3:** Properties of precursors affecting the chloride resistance of AAMs.

Year	Precursor(s)	Activators	Studied Characteristics	Improved Characteristics	Ref.
2005	MK	NaOH + Na_2_SiO_3_	Increasing particle fineness of MK	Increased compressive strength	[111]
2010	LCFA	NaOH + Na_2_SiO_3_	Different precursors (LCFA and HCFA)	Lower porosities in LCFA-based AAMs	[112]
2010	FA	NaOH/KOH/Na_2_CO_3_ + Na_2_SiO_3_	Increasing particle fineness of FA	Decreased porosity	[113]
2012	Slag + MK	NaOH + Na_2_SiO_3_	Increasing MK content	Increased chloride resistance	[114]
2013	Slag + FA	NaOH + Na_2_SiO_3_	Increasing FA content	Increased volume of permeable voids; reduced chloride penetration resistance; increased N-A-S-H gelation	[115]
2014	Slag + FA	Na_2_SiO_3_	Increasing FA content	Increased N-A-S-H gelation	[116]
2016	FA + MK	NaOH + Na_2_SiO_3_	Increasing MK content	Decreased average pore size; increased N-A-S-H gelation	[117]
2017	Slag + CLDH	Na_2_CO_3_+ Na_2_SiO_3_	Slag substituted by CLDH	Enhanced chloride binding capacity; reduced chloride ingress	[25]
2017	Slag/MK/RHS	NaOH	Increasing particle fineness	Increased compressive strength	[118]
2020	Slag + MK + dolomite	NaOH + Na_2_SiO_3_	Slag substituted by MK and dolomite	Enhanced chloride-binding capacity; decreased chloride penetration; increased hydrotalcite formation	[23]

LCFA = low-calcium fly ash; HCFA = high-calcium fly ash; CLDH = calcined layered double hydroxide.

**Table 4:** Activator compositions and silica moduli affecting the chloride resistance of AAMs.

Year	Precursor(s)	Activators	Alkali Concentration (wt. % Na_2_O/mol NaOH)	Silica Modulus	Improved Characteristics	Ref.
2012	Slag + MK	NaOH + Na_2_SiO_3_	9.1/11.6/13%	2.4	Reduced water sorptivity and chloride permeability	[114]
2013	FA	NaOH	1.0/1.3/1.5 mol	0.33/0.67/0.77/1	Reduced porosities with alkali concentration and silica modulus increasing	[108]
2013	Slag	NaOH + Na_2_SiO_3_	5/15%	0.6/1.5	Enhanced chloride attack resistance with silica modulus increasing	[36]
2014	FA	NaOH + Na_2_SiO_3_	8/10/12/14/16/18 mol	3.33	Decreased chloride penetration depth	[125]
2017	Slag	NaOH + Na_2_SiO_3_	2/4 mol	0.41/1.22	Refined pore structures with silica modulus increasing	[126]
2017	Slag + micro/nanosilica	NaOH + Na_2_SiO_3_	4 mol	2.35	Enhanced chloride penetration resistance with additional silica added	[127]
2018	Slag + nanosilica	NaOH/KOH + Na_2_SiO_3_	6 mol	2.33	Enhanced chloride penetration resistance with additional silica added	[15]
2018	Slag	NaOH + Na_2_SiO_3_	4/6/8%	0.75/1.00/1.50/ 2.00	Increased chloride binding capacity with silica modulus increasing	[128]
2019	Slag	NaOH/Na_2_CO_3_/Na_2_SO_4_/KOH/K_2_CO_3_	—	—	Highest chloride binding capacity by sulfate-activated	[18]
2019	Slag + FA	NaOH + Na_2_SiO_3_	2/4/6/8%	0.5/1.0/1.5	Decreased chloride migration coefficient, reduced porosity with alkali concentration increasing/silica modulus increasing	[106]
2020	Slag	NaOH + Na_2_SiO_3_	2–6%	2.35	Reduced chloride penetration resistance with alkali concentration increasing/ silica modulus increasing	[35]

**Table 5:** Curing conditions affecting the chloride resistance of AAMs.

Year	Precursor(s)	Activators	Curing Conditions			Improved Characteristics	Ref.
			Temperature	Time	Humidity		
2012	Slag	NaOH + Na_2_SiO_3_	60 °C	28 days	Air/humidity = 80%	Improved durability with humidity of 80%	[141]
2013	FA	NaOH	20/40 °C	7/28 days, 3/6 months	Humidity > 98%	Decreased water permeability and finer pore structures with extended curing time	[108]
2016	Slag	NaOH +Na_2_CO_3_ +Na_2_SiO_3_	(25 ± 2)/45 °C	28 days	Ambient humidity	Decreased porosity with temperature increasing	[140]
2016	LCFA	NaOH + Na_2_SiO_3_	60/75/90 °C	8/12/18/24 h	Ambient humidity	Increased chloride resistance with temperature increasing	[142]
2018	Slag	NaOH + Na_2_SiO_3_	20 ± 2 °C	28 days	Wet curing (humidity = 65%)/wet-dry curing	Lowest pore volume and porosity under wet/dry curing condition	[129]
2020	Slag	NaOH/Na_2_CO_3_/ Na_2_SO_4_/KOH/K_2_CO_3_	28/80 °C	56 days	Ambient humidity	Decreased porosity with temperature increasing	[90]
2020	Slag + MK + dolomite	NaOH + Na_2_SiO_3_	20/50 °C	28 days	Ambient humidity	Decreased compressive strength with temperature increasing	[23]
2020	Slag	NaOH + Na_2_SiO_3_	27 °C	7/28/90/180 days	Water curing	Enhanced chloride resistance and decreased porosity with extended curing time	[35]
